# Supplementary material for: Association of [18F]-FDG PET/CT-Derived Radiomic Features with Clinical Outcomes and Genomic Profiles in Patients with Chronic Lymphocytic Leukemia
Source: Diagnostics (Basel). 2025 May 19;15(10):1281. doi: 10.3390/diagnostics15101281 (PMC12110062; doi:10.3390/diagnostics15101281)
Supplement: Supplementary file 1 [file diagnostics-15-01281-s001.zip › diagnostics-3569212-supplementary.pdf]

Table S1. Robust features in CT datasets.

Features	PR_0	PR_1	p
wavelet-LLHglszmSmallAreaLowGrayLevelEmphasis	0.07±0.06	0.16±0.06	0.048
wavelet-LHLfirstorderMean	-2.28±4.54	0.94±2.73	0.002
wavelet-HLHglszmSmallAreaHighGrayLevelEmphasis	7.6±14.17	3.71±15.68	0.048

Features	TP53_0	TP53_1	p
wavelet-LLHglcmIdmn	0.96±0.02	0.95±0.02	0.031
wavelet-LLHglcmIdn	0.89±0.03	0.86±0.02	0.039
wavelet-HLHfirstorder90Percentile	15.79±8.17	12.79±8.84	0.035
wavelet-HLHfirstorderRange	108.88±126.47	62.05±45.81	0.035
wavelet-HLHglcmAutocorrelation	14.87±31.32	6.51±8.99	0.031
wavelet-HLHglcmJointAverage	3.05±2.39	2.21±1.16	0.047
wavelet-HLHglcmSumAverage	6.1±4.81	4.37±2.31	0.031
wavelet-HLHgldmHighGrayLevelEmphasis	15.41±32.06	6.94±9.56	0.035
wavelet-HLHglrlmHighGrayLevelRunEmphasis	15.47±32.14	6.99±9.71	0.043
wavelet-HLHglrlmLongRunHighGrayLevelEmphasis	43.86±93.59	16.18±19.42	0.047
wavelet-HLHglszmGrayLevelVariance	2.83±5.45	0.96±1.38	0.033
wavelet-HLHglszmHighGrayLevelZoneEmphasis	16.78±31.59	6.89±9.66	0.044
wavelet-HLHngtdmComplexity	17.88±46.48	4.92±10.91	0.031
wavelet-HLHngtdmContrast	0.05±0.04	0.1±0.04	0.031
wavelet-HHLfirstorderMedian	0.15±0.7	-0.55±0.52	0.043
wavelet-HHHfirstorderSkewness	-0.15±0.76	0.08±0.11	0.047

Features	NOTCH_0	NOTCH_1	p
wavelet-LHHglcmIdmn	0.95±0.02	0.97±0.01	0.043
wavelet-LHHgldmLowGrayLevelEmphasis	0.25±0.19	0.14±0.04	0.039
wavelet-LHHglrlmLowGrayLevelRunEmphasis	0.25±0.18	0.14±0.04	0.043
wavelet-LHHglrlmShortRunLowGrayLevelEmphasis	0.2±0.14	0.11±0.03	0.035
wavelet-HLLngtdmBusyness	6.9±9.68	22.28±19.82	0.047
wavelet-HLHglcmInverseVariance	0.49±0.03	0.52±0.02	0.006
wavelet-HLHgldmDependenceVariance	12.86±3.92	9.2±2.59	0.035
wavelet-HHHfirstorderMean	-0.06±0.24	0.22±0.22	0.006
wavelet-HHHglcmClusterShade	0±0.03	-0.04±0.04	0.005
wavelet-HHHglcmJointAverage	1.87±0.85	1.76±0.47	0.039

Features	IGVH_0	IGVH_1	p
wavelet-LLHglcmIdmn	0.96±0.02	0.95±0.02	0.031
wavelet-LLHglcmIdn	0.89±0.03	0.86±0.04	0.039
wavelet-HLHfirstorder90Percentile	15.21±8.61	14.03±10.08	0.035
wavelet-HLHfirstorderRange	94.3±98.83	83.26±113.04	0.035
wavelet-HLHglcmAutocorrelation	10.57±21.48	12.45±28.74	0.031
wavelet-HLHglcmJointAverage	2.77±1.73	2.51±2.44	0.047
wavelet-HLHglcmSumAverage	5.52±3.49	5.05±4.88	0.031
wavelet-HLHgldmHighGrayLevelEmphasis	11.04±22.2	13.02±29.42	0.035
wavelet-HLHglrlmHighGrayLevelRunEmphasis	11.11±22.4	13.08±29.44	0.043
wavelet-HLHglrlmLongRunHighGrayLevelEmphasis	28.26±43.92	37.76±95.63	0.047
wavelet-HLHglszmGrayLevelVariance	1.96±2.72	2.15±5.65	0.033
wavelet-HLHglszmHighGrayLevelZoneEmphasis	13.23±25.26	12.26±26.19	0.044
wavelet-HLHngtdmComplexity	12.94±39.05	12.48±34.29	0.031
wavelet-HLHngtdmContrast	0.05±0.04	0.1±0.04	0.031
wavelet-HHLfirstorderMedian	0.11±0.78	-0.26±0.45	0.043
wavelet-HHHfirstorderSkewness	-0.15±0.79	0.07±0.15	0.047

Table S2. Robust features in PET datasets.

Features	PR_0	PR_1	p
wavelet-LHLglszmSmallAreaLowGrayLevelEmphasis	0.06±0.1	0.01±0.03	0.045
wavelet-HLLfirstorderMean	-0.1±0.13	-0.36±0.23	0.031
wavelet-HLLfirstorderMedian	-0.03±0.48	-0.43±0.31	0.015
wavelet-HLHfirstorderMean	-0.01±0.08	0.02±0.01	0.039
wavelet-HLHfirstorderSkewness	0.24±0.45	-0.17±0.33	0.031
wavelet-HHLglszmZoneVariance	7999.93±24777.75	744.46±2100.86	0.041
wavelet-LLLfirstorderSkewness	0.1±0.47	-0.28±0.2	0.043

Features	TP53_0	TP53_1	p
wavelet-LLHgldmLargeDependenceHighGrayLevelEmphasis	269.02±150.29	153.22±58.53	0.033
wavelet-HLLglcmAutocorrelation	1.84±0.5	1.48±0.18	0.047
wavelet-HLLglrlmHighGrayLevelRunEmphasis	2.16±0.45	1.88±0.21	0.039
wavelet-HLLglrlmLowGrayLevelRunEmphasis	0.71±0.11	0.78±0.05	0.039
wavelet-HHLfirstorderKurtosis	3.12±0.52	2.49±0.35	0.004
wavelet-HHLglszmSizeZoneNonUniformityNormalized	0.45±0.07	0.58±0.16	0.033
wavelet-HHLglszmZoneVariance	8578.73±25161.92	13.86±17.13	0.046

wavelet-HHHglcmClusterProminence	0.48±0.1	0.45±0.03	0.031
wavelet-HHHglcmClusterTendency	0.48±0.09	0.45±0.04	0.043

Features	NOTCH_0	NOTCH_1	p
wavelet-LHLglcmImc1	-0.17±0.12	-0.08±0.05	0.033
wavelet-LHLglszmSizeZoneNonUniformity	1.06±0.22	2.62±2.63	0.041
wavelet-LHHglszmSizeZoneNonUniformity	1±0	2.61±2.83	0.011
wavelet-LHHglszmSizeZoneNonUniformityNormalized	0.47±0.07	0.55±0.08	0.022
wavelet-HLLglcmClusterShade	0.25±0.14	0.07±0.14	0.011
wavelet-HLLglszmHighGrayLevelZoneEmphasis	2.77±0.41	2.22±0.41	0.007
wavelet-HLLglszmLowGrayLevelZoneEmphasis	0.56±0.1	0.69±0.1	0.007

Features	IGVH_0	IGVH_1	p
wavelet-LLHgldmLargeDependenceHighGrayLevelEmphasis	263.08±157.53	178.05±71.53	0.033
wavelet-HLLglcmAutocorrelation	1.8±0.53	1.59±0.24	0.047
wavelet-HLLglrlmHighGrayLevelRunEmphasis	2.13±0.48	1.98±0.22	0.039
wavelet-HLLglrlmLowGrayLevelRunEmphasis	0.72±0.12	0.75±0.06	0.039
wavelet-HHLfirstorderKurtosis	3.13±0.5	2.51±0.38	0.004
wavelet-HHLglszmSizeZoneNonUniformityNormalized	0.45±0.08	0.55±0.12	0.033
wavelet-HHLglszmZoneVariance	9291.05±26101.59	20.57±17.84	0.046
wavelet-HHHglcmClusterProminence	0.48±0.11	0.47±0.03	0.031
wavelet-HHHglcmClusterTendency	0.48±0.09	0.46±0.05	0.043
